# Crystallization Behavior of Copolyesters Containing Sulfonates

**DOI:** 10.3390/polym16081177

**Published:** 2024-04-22

**Authors:** Zhiyong Li, Yongjing Chu, Qing Huang, Xiaopei Jin, Zhicheng Qiu, Jian Jin

**Affiliations:** 1 State Key Laboratory for Modification of Chemical Fibers and Polymer Materials, College of Materials Science and Engineering, Donghua University, Shanghai 201620, China; lizhiyong@cta.gt.cn (Z.L.); cyj2380366079@163.com (Y.C.); 2 State Key Laboratory of Biobased Fiber Manufacturing Technology, China Textile Academy Co., Ltd., Beijing 100025, China; jinxiaopei@cta.gt.cn (X.J.); qiuzhicheng1@cta.gt.cn (Z.Q.); jinjian@cta.gt.cn (J.J.)

**Keywords:** sulfonate copolyester, sodium isophthalic acid-5-sulfonate, non-isothermal crystallization, ion agglomeration

## Abstract

The polar sulfonate groups in cationic dyeable polyester (CDP) lead to complex crystallization behavior, affecting CDP production’s stability. In this study, cationic dyeable polyesters (CDP) with different sulfonate group contents were prepared via one-step feeding of sodium isophthalic acid-5-sulfonate (SIPA), terephthalic acid (PTA), and ethylene glycol (EG). The non-isothermal crystallization behavior of these copolyesters was analyzed by differential scanning calorimetry (DSC). Results show that the crystallization temperature of the sample shifts to lower values with the increase in SIPA content. The relaxation behavior of the molecular chain is enhanced due to the ionic aggregation effect of sulfonate groups in CDP. Therefore, at low cooling rates (2.5 °C/min and 5 °C/min), some molecular chain segments in CDP are still too late to orderly stack into the lattice, forming metastable crystals, and melting double peaks appear on the melting curve after crystallization. When the cooling rate increases (10–20 °C/min), the limited region of sulfonate aggregation in CDP increases, resulting in more random chain segments, and a cold crystallization peak appears on the melting curve after crystallization. The non-isothermal crystallization behavior of all samples was fitted and analyzed by the Jeziorny equation, Ozawa equation, and Mo equation. The results indicate that the nucleation density and nucleation growth rate of CDP decrease with the increase in SIPA content. Meanwhile, analysis of the Kissinger equation reveals that the activation energy of non-isothermal crystallization decreases gradually with the increase in SIPA content, and the addition of SIPA makes CDP crystallization more difficult.

## 1. Introduction

Polyethylene terephthalate (PET) molecular chains have both semi-aromatic and semi-aliphatic structural characteristics, and some amorphous entangled chains are still retained after crystallization of the melt [1], resulting in good physical and mechanical properties of PET and easy processing. Thus, it is widely used in various resin products, plastics, and fiber products [2,3]. Since PET was successfully developed in the 1940s, it has become the largest synthetic fiber raw material, and its output has increased year by year [4]. However, due to the structured molecular chain structure of PET, the lack of dyeing functional groups, and the high crystallinity [5,6], the dyeing performance of PET fiber is poor, and only dispersive dyes can be dyed under high temperature and high pressure [7]. In 1958, DuPont added sodium dimethyl isophthalate-5-sulfonate (SIPM) containing sulfonate groups to the polymerization of conventional polyester and successfully synthesized cationic dyeable polyester containing sulfonate groups (CDP) [8], successfully improving the dyeing properties of conventional PET. Compared with dispersible dyes, CDP can be dyed under atmospheric pressure by cationic dyes [9]. The combination of cationic dyes and CDP is firm, and the dye has lower thermal mobility. In the CDP dyeing process, less waste liquid is produced, the energy consumption is lower, the cost is lower, and the prepared product is more colorful [10]. The third monomer SIPM was developed according to the route of CDP preparation by the DMT method. In 1956, the United States Mid-Century Company, the United Kingdom ICI Company, etc., applied for the patent of purified terephthalic acid (PTA) [11] and continuous production. Subsequently, the PTA process gradually replaced the DMT process to produce PET. In 1970, Toray changed the third monomer for preparing CDP from SIPM to SIPE (sodium-5-sulfo-bis-(hydroxyethyl)-isophthalate) [12], to better adapt to the direct esterification route of PTA. Then, the route for preparing CDP by the SIPE method was gradually widely used [13]. However, in the process of transesterification preparation of SIPE by SIPM and EG, autopolymerization is prone to occur, resulting in excessive by-products [14], thus affecting the subsequent CDP preparation process and product quality [15]. To reduce SIPE self-polymerization and other side reactions, it is necessary to use process control, such as diluting it with ethylene glycol, controlling the preparation temperature of SIPE, storage temperature, reaction temperature during addition, etc., which makes the process of preparing CDP by the SIPE method produce more energy consumption and cost. To avoid these problems, the preparation of cationic dyeable polymers by the SIPA process has been studied at home and abroad [15,16].

SIPA (sodium isophthalic acid-5-sulfonate) is the initial raw material for the production of SIPM and SIPE. If SIPA is directly used as the raw material for the preparation of copolyesters containing sulfonates, the one-step feeding reaction can save the preparation process of SIPM and SIPE and reduce energy consumption and cost [17]. In the process of polycondensation, SIPA reacts with EG to shrink water, and the reaction component is singular, which avoids the situation of insufficient stability in production. At present, there is little research on the crystallization and melting behavior of CDP at home and abroad. To investigate the melting and crystallization behavior of such ionomers, Eisenberg-Hird-Moore et al. [18] proposed the ionomer cluster model, for which it was believed that ion aggregation would restrict the movement of surrounding molecular chains, thus forming restricted regions. With the increase in ion content, these restricted regions would gradually overlap, thus forming ion aggregation clusters, increasing the physical entanglement between molecular chains [19] and then affecting the melting and crystallization behavior of the polymer [20]. Jun Wang et al. [13] compared the thermal and crystallization properties of CDP, ECDP, and MCDP containing different copolymerization components. They found that the sodium sulfonate ionic groups in them would react with the ester bond on the PET molecular chain. The resulting sodium carboxylate chain end could evolve into the crystal nuclei, thereby enhancing its cold crystallization ability. Yue Yin et al. [21] prepared PET ionomers (PETi) with different sulfonated 1, 4-butanediol (SBDO) contents through melt polycondensation. They found that due to the agglomeration of sulfonate ions in SBDO, SBDO became the nucleating agent for PETi crystallization, avoiding the deterioration of PET mechanical properties caused by the excessive addition of traditional nucleating agents. Zheng Huang et al. [22] studied the thermal properties and rheological properties of CDP modified by SIPE with different contents and found that when the content of SIPE exceeded 3 mol%, sulfonate ions in CDP formed ion aggregation clusters, thus affecting the melting and rheological behavior of molecular chains in the melt. It can be seen that the ionic agglomeration effect of sulfonate groups in CDP has a great influence on the crystallization and melting behavior of the polymer, which makes the properties and processing technology of CDP need targeted control. Since industrial CDP production is still dominated by the SIPE method, there is little research on the melting and crystallization behavior of CDP directly prepared by SIPA. In this work, SIPA was directly used to prepare CDP with different SIPA contents. Through non-isothermal crystallization experiments, the influence of sulfonate groups on the crystallization and melting behavior of the CDP molecular chain was studied, to guide the development and application of a new CDP process.

## 2. Materials and Methods

### 2.1. Materials and Synthesis

#### 2.1.1. Materials

PTA: industrial grade, Hengli Petrochemical (Dalian, China) Co., Ltd. EG: industrial grade, produced by Zhongsha (Tianjin, China) Petrochemical Co., Ltd. SIPA: industrial grade (content ≥ 99%), produced by Shandong Dekang Chemical Co., Ltd. (Dezhou, China). Ethylene glycol antimony: industrial grade, Tonggu County, Jiangxi Province, Eryuan Chemical Co., Ltd. (Yichun, China).

#### 2.1.2. Synthesis

**Figure 1:**
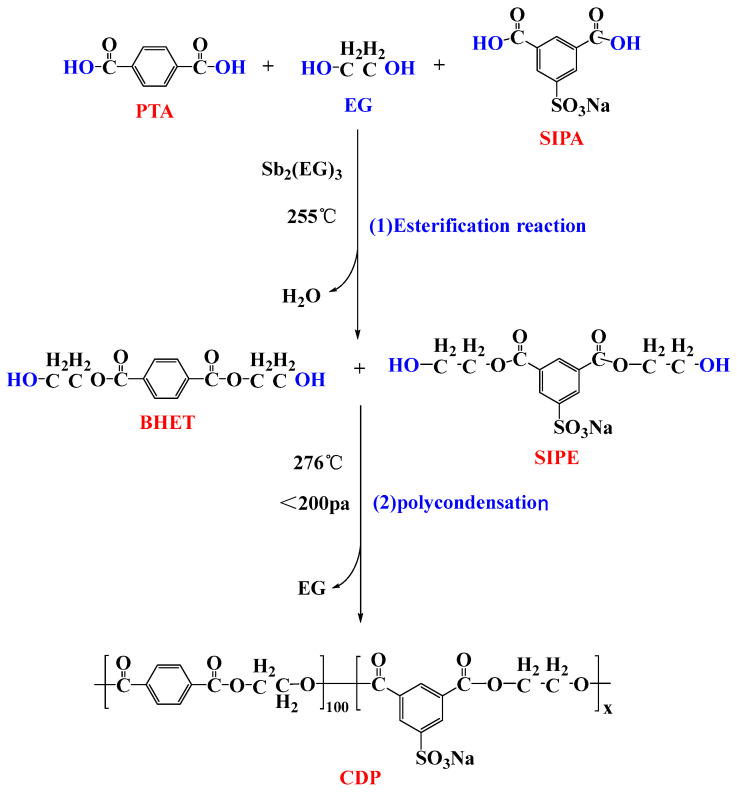
The synthesis process of CDP, where x represents the molar fraction of SIPA in 100 molPTA (SIPA: PTA = x:100 mol).

PET and CDP copolyesters with different SIPA contents were prepared by a one-step feeding method, as shown in Figure 1 below. Reaction components such as sodium isophthalic acid-5-sulfonate (SIPA), terephthalic acid (PTA), ethylene glycol (EG), and catalyst glycol antimony were combined according to the molar ratio of alcohol to acid 1.4:1. We placed it into a 30 L polymerization kettle (made by Yangzhou Huitong Chemical Technology Co., Ltd., Yangzhou, China) for stirring and mixing and gradually heated it to 255 °C for esterification reaction. When the esterification dehydration reached more than 90%, the temperature rose to 276 °C for polycondensation reaction, the vacuum was controlled below 200 pa, and the material was discharged under the same stirring current to obtain copolyesters with different sodium sulfonate group contents. According to proportions of SIPA in PTA molar number of 0%, 1.5%, and 3.0%, the sample names were recorded as S0, S1.5, and S3.0, respectively.

### 2.2. Characterization

#### 2.2.1. Intrinsic Viscosity

The intrinsic viscosity ([*η*]/dL·g^−1^) of the polymer was measured in a water bath at 25 °C using an Ulster viscometer (NCY, Shanghai SIERDA Scientific instrument Co., Ltd., Shanghai, China). The polymer was dissolved in a phenol/1, 1, 2, 2-tetrachloroethane solution with a mass ratio of 1∶1. The test of [*η*] was repeated at least three times to ensure that the error of residence time was within 0.5 s.

#### 2.2.2. Non-Isothermal Crystallization

The kinetics of non-isothermal crystallization were analyzed by differential scanning calorimetry (DSC8000 differential scanning calorimeter, Perkin-Elmer, Waltham, MA, USA). In the first step, under an atmosphere of nitrogen, the prepared sample was heated from 30 °C to 280 °C at a rate of 80 °C/min and held for 5 min, to eliminate the thermal history. Secondly, we cooled the samples to 30 °C at different cooling rates (40 °C/min, 20 °C/min, 10 °C/min, 5 °C/min, 2.5 °C/min). Finally, the samples were heated to 280 °C again at a rate of 20 °C/min. The data of the cooling curve and the second heating curve under different cooling rates were analyzed.

## 3. Results and Discussion

### 3.1. Intrinsic Viscosity

Table 1 exhibits the intrinsic viscosity values of S0, S1.5, and S3.0. With the increase in SIPA content, the intrinsic viscosity of CDP gradually decreases. This is because the asymmetric structure of SIPA can produce steric hindrance [23], and at the same time, the sulfonate ionic groups in SIPA can produce ion clusters in intermolecular association, resulting in increased melt viscosity of the system, limited molecular chain migration [24], and hindering the increase in molecular weight. In this polymerization process, the reaction was terminated by controlling the system at the same melt viscosity value. With the increase in SIPA content, the melt viscosity increases faster, and the intrinsic viscosity ([*η*]) of the product decreases gradually.

### 3.2. Non-Isothermal Crystallization

#### 3.2.1. Non-Isothermal Crystallization Behavior of CDP at Different Cooling Rates

**Figure 2:**
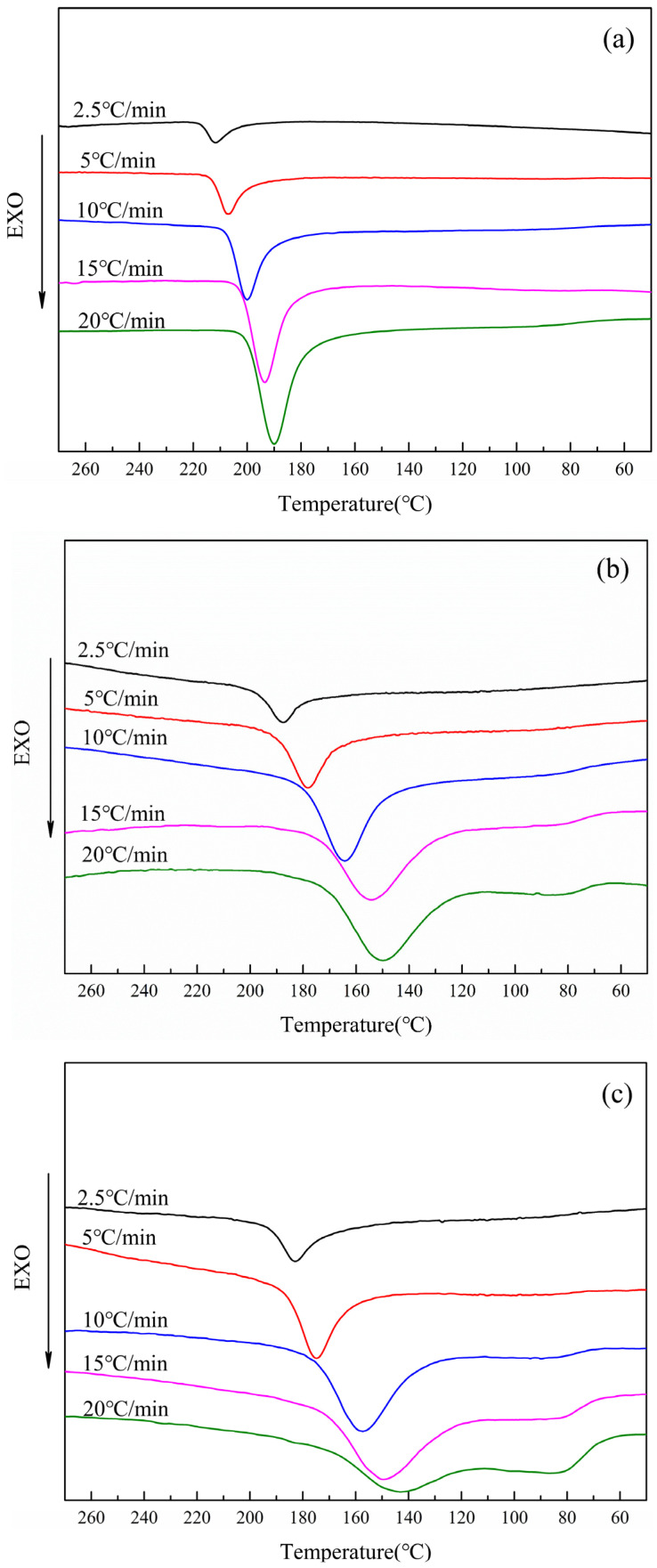
Non-isothermal crystallization curves of PET and CDP with different SIPA contents: (**a**) S0; (**b**) S1.5; (**c**) S3.0.

The non-isothermal crystallization curves of PET and CDP with different SIPA contents are shown in Figure 2. The peak crystallization temperature ($T_{P}$), the initial crystallization temperature ($T_{b}$), the crystallization ending temperature ($T_{e}$), and the hot crystallization enthalpy value (${△H}_{mc}$) can be obtained by DSC cooling curves, as shown in Table 2 below. The crystallization rate of each sample was judged according to the crystallization rate coefficient ORC proposed by Y. P. KHANNA et al. [25,26] (Equation (1)), and the ORC is listed in Table 2.

(1) $$ORC=△\phi /△T_{P}$$

where ϕ is the cooling rate of non-isothermal crystal, °C/min; $T_{P}$ is the peak temperature of non-isothermal crystallization, °C; and ORC is the crystallization rate coefficient, which is inversely proportional to the non-isothermal crystallization rate, min^−1^.

**Figure 3:**
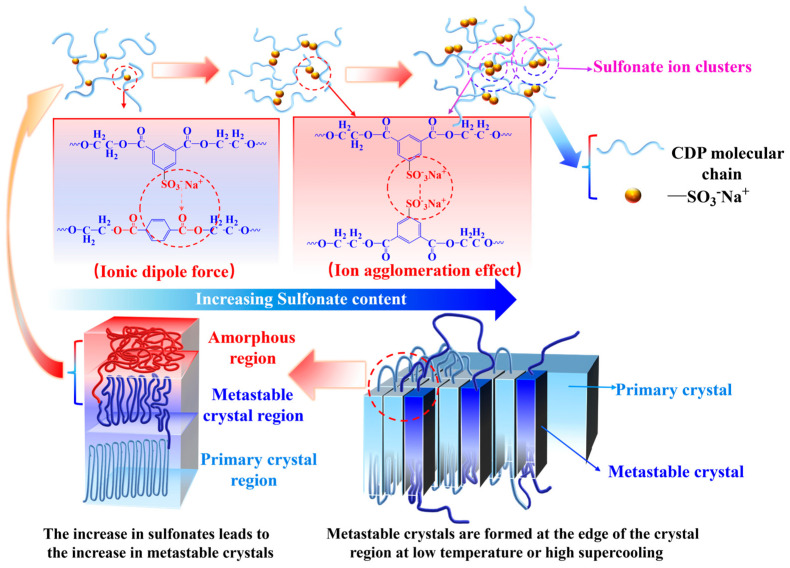
Influence of sulfonate ion agglomeration effect on the non−isothermal crystallization of CDP molecular chains in CDP.

It can be seen from Table 2 that the value of ORC increases with the increase in SIPA content, indicating that the non-isothermal crystallization rate of the sample decreases with the increase in SIPA content. The content of sulfonate groups in the molecular chain increases with the increase in SIPA content, resulting in the enhancement of its hindrance to the molecular chain and the decrease in the non-isothermal crystallization rate. It can be seen from Figure 2 and Table 2 that at the same cooling rate, the crystallization peaks of S0, S1.5, and S3.0 gradually widen and the crystallization peak temperature $T_{P}$ gradually decreases. This is because the introduction of SIPA into PET destroys the regularity of the molecular chain. At the same time, the ionic dipole force generated between the sulfonate ion and the ester oxygen atom in the molecular chain increases the intermolecular chain force, resulting in an enhanced relaxation effect of the polymer molecular chain. The molecular chain needed more time to adjust its conformation and then was released into the lattice. The crystallization peak widths and $T_{P}$ decreased. With the increase in SIPA content, the concentration of sulfonate ions in the CDP molecular chain increases, and the restricted region formed by the ionic aggregates generated between sulfonate ions gradually increases [27], further hindering the migration of molecular chains, resulting in the extension of non-isothermal crystallization time, a wider crystallization peak, and lower $T_{P}$. The limiting effect of sulfonate groups on the non-isothermal crystallization of CDP molecular chains is shown in Figure 3 below [22,28,29,30].

It can also be seen in Figure 2 that under the cooling rates of 10 °C/min, 15 °C/min, and 20 °C/min, an obvious glass transition exists on the cooling curves of S1.5 and S3.0, while it does not appear on the cooling curves of S0. This is because the addition of SIPA increases the relaxation time of CDP molecular chain motion, resulting in imperfect crystallization of CDP at a higher cooling rate. First of all, when the cooling rate is low (2.5 °C/min, 5 °C/min), the driving force of crystallization brought by the cooling rate is small, the formation of crystal nuclei is reduced, and the molecular chain has more time to discharge into the lattice, resulting in perfect crystallization and larger spherulite size [31]. However, when the cooling rate is high (10 °C/min, 15 °C/min, 20 °C/min), the driving force of crystallization increases and the number of crystal nuclei formed in CDP increases. However, due to the relaxation effect, the fluidity of the molecular chain is limited, the degree of crystallization is reduced, and the size of the spherulites formed is small. When the temperature is reduced to near the glass transition temperature, because there are more molecular chain segments that do not enter the lattice in the random region of CDP, these molecular chain segments form a more obvious glass transition on the cooling curve when they are frozen.

#### 3.2.2. Variation in Relative Crystallinity (*X*(*T*)) with Temperature (*T*)

Relative crystallinity $X\left(T\right)$ is the integral of the exothermic crystallization peak in the DSC cooling curve at unit temperature (dT). It is assumed that the heat released during crystallization has a linear relationship with relative crystallinity $X\left(T\right)$ at temperature T (Equation (2)) [32].

(2) $$X\left(T\right)=\frac{\int_{T_{0}}^{T}\left(\frac{dH_{c}}{dT}\right)dT}{\int_{T_{0}}^{T_{\infty }}\left(\frac{dH_{c}}{dT}\right)dT}$$

where $X\left(T\right)$ is the relative crystallinity at temperature T, %; $T_{0}$ represents the initial crystallization temperature, °C; $T_{\infty }$ represents the crystallization termination temperature, °C; and $dH_{c}$ is the enthalpy change at an infinitesimal temperature interval dT (J/g).

**Figure 4:**
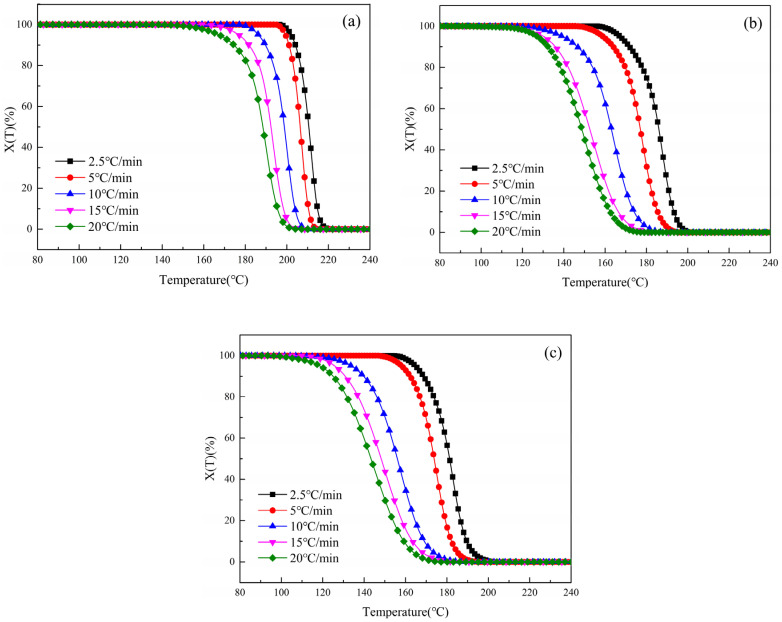
Curves of *X*(*T*) of PET and CDP with different SIPA contents as a function of temperature *T* at different cooling rates: (**a**) S0; (**b**) S1.5; (**c**) S3.0.

According to Equation (1), the temperature-rise data of non-isothermal crystal DSC has been processed, and the curve of $X\left(T\right)$ with temperature T can be obtained, as shown in Figure 4 below. It can be seen from Figure 4 that at each cooling rate, the beginning and end crystallization temperatures of S1.5 and S3.0 are lower than those of S0, which is consistent with the previous cooling curve analysis, indicating that the addition of SIPA disrupts the regularity of CDP molecular chains and the ion aggregation effect caused by polar sulfonate groups and even hinders the orderly arrangement of CDP molecular chains. As a result, S1.5 and S3.0 require lower crystallization temperatures and longer crystallization times.

#### 3.2.3. Variation in Relative Crystallinity (*X*(*t*)) with Time (*t*)

In the non-isothermal crystallization process, the crystallization temperature T can be converted into time *t* by the Equation (3).

(3) $$t=\frac{\left(T_{0}-T\right)}{\phi }$$

where t is time, min; and ϕ is cooling rate, °C/min. $X\left(t\right)$ can be obtained by conversion according to Equations (2) and (3). See Equation (4) below.

(4) $$X\left(t\right)=\frac{\int_{t_{0}}^{t}\left(\frac{dH_{c}}{dt}\right)dt}{\int_{t_{0}}^{t_{\infty }}\left(\frac{dH_{c}}{dt}\right)dt}$$

where $X\left(t\right)$ is the relative crystallinity at time *t*, °C; $t_{0}$ represents the initial crystallization time, $t_{\infty }$ represents the crystallization end time, min; and $dH_{c}$ is the enthalpy change at an infinitesimal temperature interval dt, J/g.

**Figure 5:**
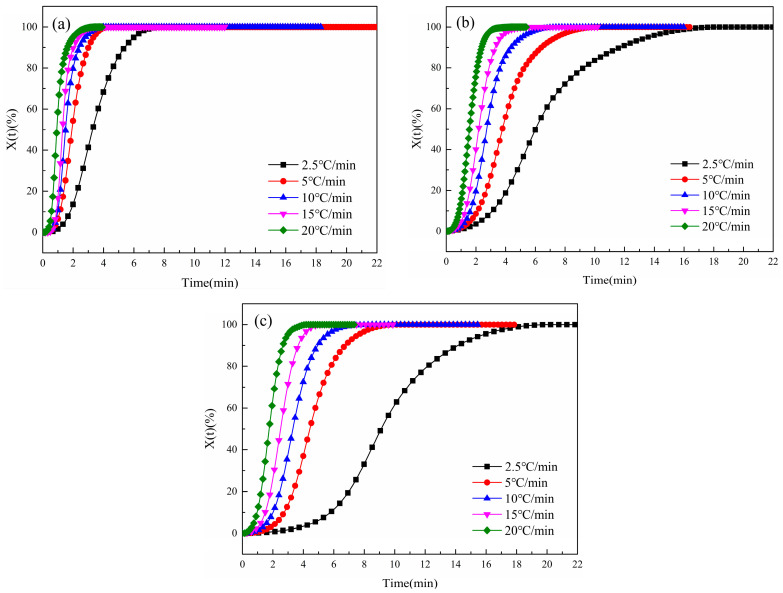
Curves of *X*(*t*) of PET and CDP with different SIPA contents as a function of temperature t at different cooling rates: (**a**) S0; (**b**) S1.5; (**c**) S3.0.

According to Equation (4), the DSC data of non-isothermal crystallization have been processed, and the curves of $X\left(t\right)$ with time t have been obtained, as shown in Figure 5 below. It can be seen from Figure 5 that the $X\left(t\right)$ curve of the sample with time t forms an “S” shape, which is a typical sigmoid curve [33]. This shape of the curve is related to the relaxation of the polymer chain, and the three sections of the curve respectively represent the generation of the crystal nuclei, the growth of crystal nuclei, and the termination of crystallization [34]. At the initial stage of crystallization, when the temperature drops below the melting point, the fluctuation in the local free energy of the polymer causes the molecular chain to spontaneously fold and curl, due to the minimum law of free energy [35], resulting in the initial “baby nuclei”. As the temperature continues to decrease, under the influence of thermodynamics, to reduce the surface energy inside the polymer, the molecular chains continue to stack towards the crystal nuclei, resulting in the growth of the crystal nuclei [36]. In CDP, the polar sulfonate groups not only increase the number of random chain segments but also increase the interchain force, resulting in increased relaxation time of the molecular chain. Therefore, compared with conventional PET, the stacking speed of CDP molecular chains is slower, and the entire crystallization process takes more time. With the growth of crystal nuclei, spherulites are gradually formed. Due to the limited space, spherulites begin to squeeze and collide with each other, resulting in changes in the stacking mode of molecular chains between spherulites. Some molecular chain segments are not completely crystallized and become metastable crystals. There are fewer ordered crystals in CDP, and more molecular segments enter the metastable region, due to the structure of SIPA and the influence of sulfonate groups.

#### 3.2.4. Melting Temperature Curve of CDP at Different Cooling Rates

**Figure 6:**
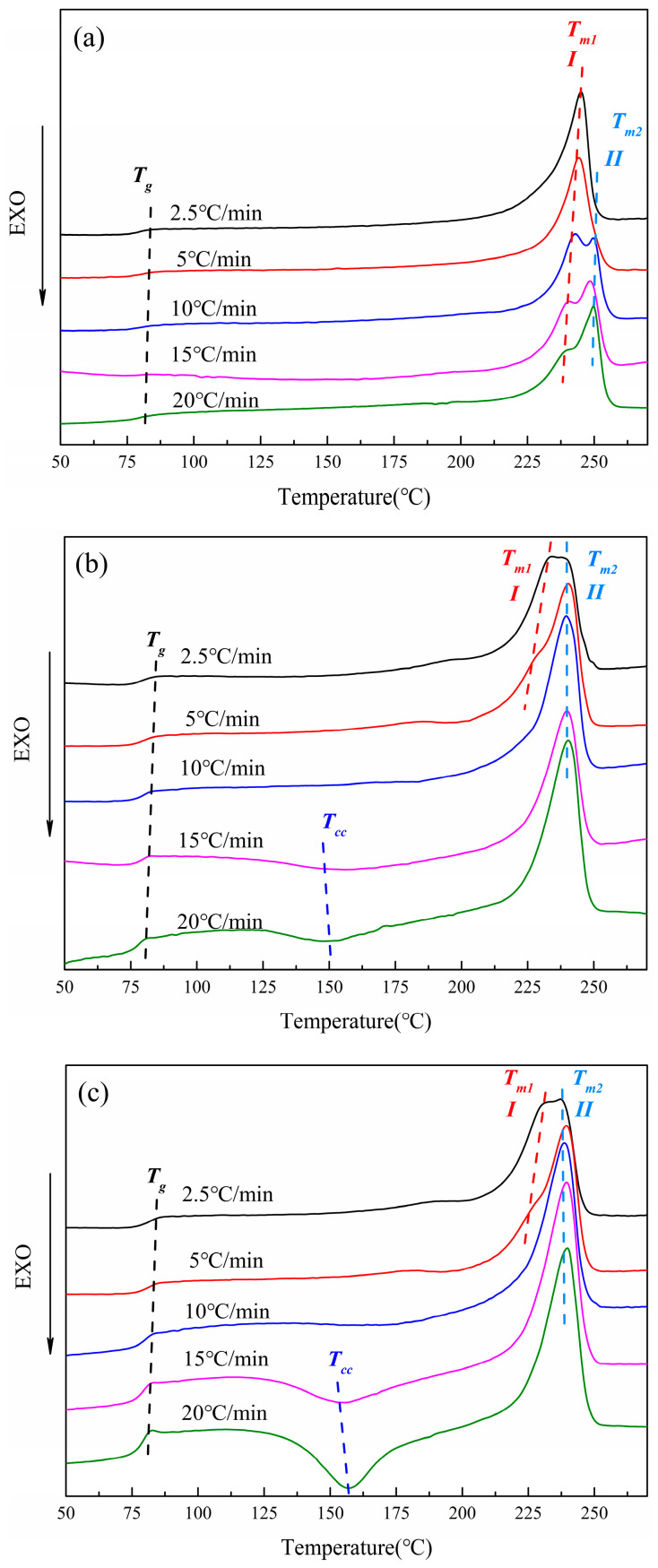
CDP melting temperature curves of PET and different SIPA contents: (**a**) S0; (**b**) S1.5; (**c**) S3.0.

Figure 6 below shows the temperature melting curves of PET and CDP with different SIPA contents. The glass transition temperature ($T_{g}$), cold crystallization peak temperature ($T_{cc}$), cold crystallization enthalpy value (${△H}_{cc}$), peak melting temperature ($T_{m1}$, $T_{m2}$), and melting enthalpy value (${△H}_{m}$) are shown in Table 3 below. As can be seen from Figure 6a, when the cooling rate of S0 reaches 10 °C/min, the temperature-rise melting peak after non-isothermal crystallization produces a double peak. With the increase in cooling rate, the intensity of low-temperature peak *I* gradually weakens, while that of high-temperature peak *II* gradually increases, with little change in peak value.

The endothermic double peaks on the melting curve are caused by the secondary crystallization behavior of the polymer molecular chain [37] which, on the one hand, thickens the primary crystal and, on the other hand, leads to the rearrangement of some metastable crystals to form new crystals [38]. The low-temperature endothermic peak *I* in Figure 6a represents the melting of the primary crystal, and the high-temperature endothermic peak *II* represents the melting of the thickening part of the primary crystal and the melting of the partial metastable crystal rearrangement to form the crystal [39,40]. At each cooling rate, the secondary crystallization behavior of the polymer molecular chain is different according to the size of the crystallization driving force. At a lower cooling rate (2.5, 5 °C/min), the molecular chains in the metastable crystals during the melting process are more inclined to pile up on the surface folds of the primary crystals due to the smaller crystallization driving force, and the primary crystals are more perfect. In the subsequent melting curve, the enthalpy generated by the melting of the primary crystal accounts for the main part, and only the endothermic single peak *I* appears. With the increase in cooling rate (10–20 °C/min), the driving force of crystallization increases, and the number of crystal nuclei increases during crystallization. At the same time, the increase in cooling rate leads to more molecular chain segments unable to be discharged into the lattice, an increase in metastable crystals, and a decrease in spherulite size. In the subsequent melting process, due to the increase in metastable crystals, the secondary crystallization behavior of molecular chains is enhanced, and more secondary crystals are formed [35]. The melting of secondary crystals and the thickened part of primary crystals form endothermic peak *II*, resulting in the melting double-peak phenomenon. This phenomenon gradually increases with the increase in the cooling rate, resulting in the gradual enhancement of endothermic peak *II* and the gradual weakening of endothermic peak *I*.

As shown in Figure 6b,c, with the increase in cooling rate, the intensity of low-temperature endothermic peak *I* on the melting curves of S1.5 and S3.0 gradually weakens, while the intensity of high-temperature endothermic peak *II* gradually increases. When the cooling rate reaches 10 °C/min, the endothermic peak *I* begins to disappear. At the same time, when the cooling rate is 15 °C/min and 20 °C/min, an obvious cold crystallization peak can be seen in Figure 6b,c. With the increase in the cooling rate, the glass transition on the temperature curve of S1.5 and S3.0 becomes more obvious. As mentioned above, in Figure 6b,c, the low-temperature endothermic peak *I* represents the melting of the primary crystal, and the high-temperature endothermic peak *II* represents the melting of the secondary crystal formed by the thickening part of the primary crystal during the secondary crystallization process and the partial molecular chain rearrangement in the metastable crystal. The secondary crystallization behavior is greatly affected by metastable crystals during melting temperature rise. In CDP, the irregularity of molecular chains is enhanced due to the introduction of SIPA. At the same time, the ionic dipole force between sulfonate groups and molecular chains hinders the movement of molecular chains, and the relaxation effect of molecular chains is enhanced, resulting in difficult molecular chain crystallization. In the non-isothermal crystallization process, compared with PET, even at a lower cooling rate (2.5, 5 °C/min), the CDP molecular chain still does not have enough time to adjust the conformation into the lattice, resulting in imperfect spherulites and an increase in metastable crystals. In the subsequent heating process, the secondary crystallization behavior of the molecular chain is enhanced, and more secondary crystals are formed. The melting of the secondary crystal and the thickened part of the primary crystal forms endothermic peak *II*, resulting in the melting double-peak phenomenon. As with PET, this phenomenon gradually increases as the cooling rate increases, resulting in a gradual enhancement of endothermic peak *II* and a gradual weakening of endothermic peak *I*.

When the cooling rate is increased to 10–20 °C/min, the relaxation effect of the CDP molecular chain is enhanced, the crystallization of the CDP molecular chain is more inadequate, the perfection of crystallization is further reduced, and the random chain segments are increased. In the subsequent melting process, the activity of random molecular segments is enhanced, and the random segments in some metastable crystals are rearranged at $T_{cc}$, resulting in cold crystallization. At the same time, it can be seen from Figure 6b,c that with the increase in SIPA content, the glass transition and cold crystallization peak of CDP in the heating process are more obvious at the cooling rates of 15 °C/min and 20 °C/min, which indicates that with the increase in sulfonate content, the restricted region of molecular chains caused by sulfonate ion accumulation expands [41]. Its obstruction effect on molecular chain migration is enhanced, resulting in difficulty in CDP molecular chains’ bonding into the lattice and more random chain segments, and the glass transition and cold crystallization peak are more obvious when the temperature is raised again.

#### 3.2.5. Jeziorny Method

The classical Avrami equation is often used to describe the primary stage of polymer isothermal crystallization kinetics, as shown in Equation (5) [42].

(5) $$X\left(t\right)=1-exp(-Zt^{n})$$

where $X\left(t\right)$ is the relative crystallinity, %; n is the Avrami index, which is a parameter related to crystallization mechanism and nucleation type [43]; and Z is the crystallization rate constant, which is related to nucleation density and nucleation growth rate, min^−n^.

However, this model is not suitable to describe the non-isothermal crystallization kinetics of polymers. According to Jeziorny, the initial stage of non-isothermal crystallization can be simplified by the Avrami equation at a constant cooling rate, and the non-isothermal crystallization process can be decomposed into an infinite number of isothermal crystallization processes at different temperatures [44]. The parameter $Z_{t}$ in the Avrami equation is modified to the non-isothermal crystallization parameter $Z_{c}$ (Equation (6)).

(6) $$lgZ_{c}={lgZ}_{t}/\phi$$

where $Z_{t}$ is the isothermal crystallization rate constant, which is related to nucleation density and nucleation growth rate, min^−n^; $Z_{c}$ is a non-isothermal crystallization rate constant and has the same physical meaning as $Z_{t}$; and ϕ is the cooling rate, min^−1^.

**Figure 7:**
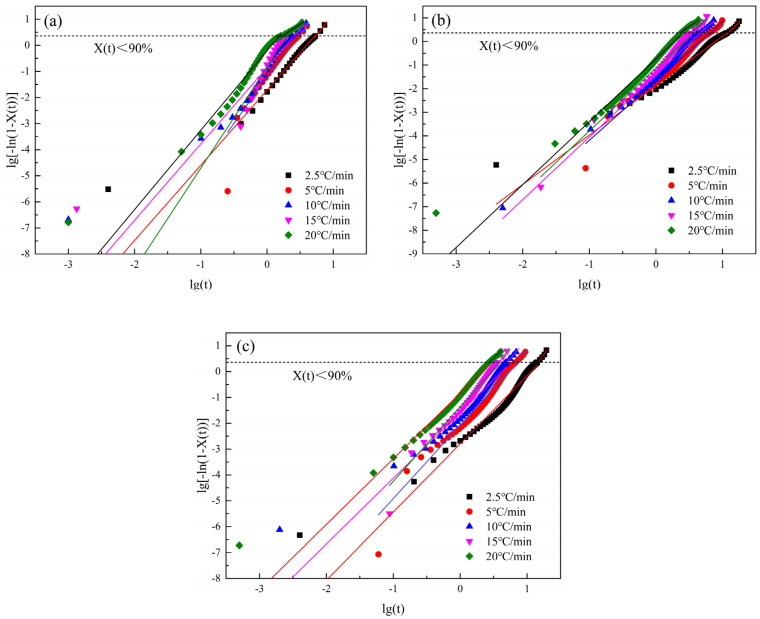
Relationship between lg[−ln(1 − *X*(*t*))] and lg*t* at different crystallization cooling rates. (**a**) S0; (**b**) S1.5; (**c**) S3.0. (The dotted lines in the figure represent the polymer at 90% relative crystallinity.)

We substitute Equation (6) into Equation (5) to obtain Equation (7).

(7) $$lg\left[-ln\left(1-X\left(t\right)\right)\right]=lgZ_{c}+nlgt$$

where t is time, min; $X\left(t\right)$ is the relative crystallinity, %; and n is the apparent Avrami index, which has no physical significance [45]. Data when $X\left(t\right)$ is greater than 90% and data when $X\left(t\right)$ is less than 90% was substituted into the Jeziorny and linear fitting was carried out, as shown in Figure 7, to obtain $n_{1}$, $Z_{c1}$ and $n_{2}$, $Z_{c2}$. The data are listed in Table 4.

It can be seen from Figure 7 that the data of S0, S1.5, and S3.0 are all in a straight-line shape, which basically conforms to the Jeziorny equation. Combined with the data in Table 4, it can be seen that when $X\left(t\right)$ is greater than 90%, the linear fitting correlation coefficient R_1_^2^ is greater than the linear correlation coefficient R_2_^2^ when $X\left(t\right)$ is less than 90%. This indicates that when PET and CDP crystal nuclei gradually grow to form spherulites, in a certain space, spherulites squeeze and collide with each other, resulting in changes in their crystallization mode [46]. Therefore, when $X\left(t\right)$ is greater than 90%, the change law of their data gradually deviates from the Jeziorny equation. It can also be seen from Figure 7 that the fit degree of the overall data of S0 to the Jeziorny equation increases with the increase in the cooling rate, while the fit degree of the overall data of S1.5 and S3.0 to the Jeziorny equation decreases with the increase in the cooling rate.

**Figure 8:**
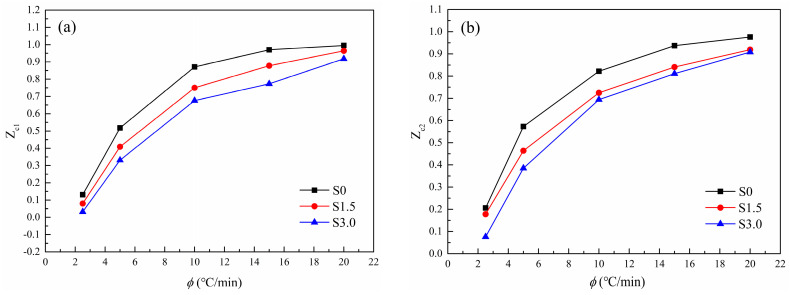
Curves of non−isothermal crystallization kinetics parameters of PET and CDP with different SIPA contents: (**a**) $Z_{c1}$; (**b**) $Z_{c2}$.

It can be seen from Table 4 that the n of PET and CDP is between 2.0 and 4.0, indicating that the crystal growth dimension of PET and CDP non-isothermal crystals is between two and three dimensions, which accords with the crystal growth characteristics of conventional polyester. Rate constant curves ($Z_{c1},Z_{c2}$) are shown in in Table 4 with *ϕ* and in Figure 8. In Figure 8, you can see that the $Z_{c1}$ and $Z_{c2}$ values of S0, S1.5, and S3.0 all gradually increase with increasing *ϕ*, indicating that the crystallization rate of PET and CDP decreases with increasing *ϕ*. This is because with increasing *ϕ*, the crystalline driving force of the PET and CDP molecular chains increases and the nuclear density increases, making the *Z* values of S0, S1.5, and S3.0 increase with increasing *ϕ*. The *Z* values of S0, S1.5, and S3.0 for the same *ϕ* gradually decrease, indicating that the crystallization rate of CDP decreases with the increase in SIPA content. In the CDP molecular chain, the crystallization rate of PET at each *ϕ* is higher than CDP because SIPA destroys the orderliness of the CDP molecular chain and the limitation of CDP molecular chain movement by the sulfonate ionic aggregates makes the CDP molecular chains’ bonding into the lattice take longer. Moreover, with the increase in SIPA content, the concentration of sulfonate ion aggregates in CDP increases, resulting in the expansion of the restricted region of molecular chains caused by sulfonate ion aggregation [41], and the resistance to molecular chain migration increases, resulting in a decrease in the crystallization rate of CDP.

#### 3.2.6. Ozawa Method

Under non-isothermal conditions, crystallization becomes very complicated. The Avrami model is based on the growth process of crystal nuclei simulated by constant nucleation and growth rate. The Avrami model is an isotropic crystal growth model under ideal conditions and is not suitable for non-isothermal crystal conditions with constant temperature changes. However, Jeziorny adjusted the Avrami model and proposed the Jeziorny equation which was too rough. The Avrami index was called the apparent Avrami index, which has no specific physical significance and cannot explain the changes in the size of the crystal nuclei and the nucleation mode during crystallization. In this regard, the Ozawa model proposed by Ozawa takes the cooling rate as a variable given the continuous temperature changes in the non-isothermal crystallization process, and the Ozawa index has clear physical significance (Equation (8)).

(8) $$1-X\left(T\right)=exp(-k(T)/\phi^{m})$$

where $X\left(T\right)$ is the relative crystallinity, %; ϕ is the cooling rate, °C/min; and m is the Ozawa index, which is related to crystallization mechanism and nucleation type. k(T) is a temperature function related to nucleation rate, nucleation mode, and nucleation growth rate, (K/min)^m^.

Take the logarithm of both sides of Equation (8) to obtain Equation (9).

(9) $$lg\left[-ln\left(1-X\left(T\right)\right)\right]=lgk\left(T\right)-mlg\phi$$

**Figure 9:**
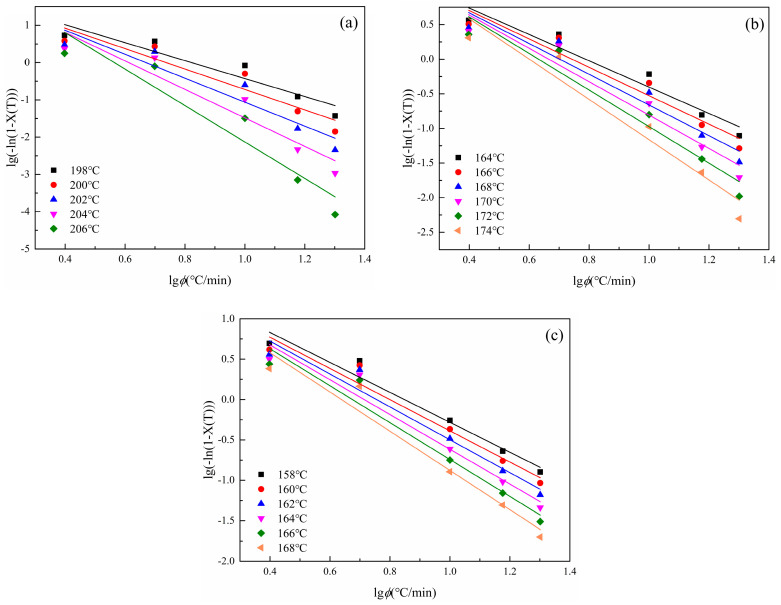
Relationship between lg[−ln(1 − *X*(*T*))] and lg*ϕ* at different crystallization cooling rates. (**a**) S0; (**b**) S1.5; (**c**) S3.0.

Since the Ozawa model cannot fully cover the entire crystallization process, six evenly spaced temperature points were selected, with lgϕ as the *X* axis and $lg\left[-ln\left(1-X\left(T\right)\right)\right]$ as the *Y* axis, and linear fitting was performed, as shown in Figure 9, and m and k(T) can be obtained. The data are listed in Table 5.

Figure 9 shows the curve obtained by fitting the non-isothermal crystallization data into the Ozawa model. Combined with the linear correlation coefficient R^2^ in Table 5, it can be seen that the non-isothermal crystallization data of S0 at all temperatures have a lower degree of agreement with the Ozawa model than S1.5 and S3.0. The m and k(T) values of S0 cannot be compared with those of S1.5 and S3.0. This is due to the narrow crystallization temperature range of PET at the selected crystallization rate, and the overall crystallization of S0 cannot be described in the Ozawa equation, resulting in the low compatibility of S0 to the Ozawa equation at various temperatures [47]. Moreover, at different temperatures, the curve fitted by the Ozawa equation has a large deviation, and the comparison between S0 and the parameters of S1.5 and S3.0 regarding the Ozawa equation has no reference value. Therefore, the Ozawa method does not apply to polymers with different crystallization processes [47], which is consistent with the results of previous studies [48].

#### 3.2.7. Mo Method

The Avrami equation is a model to describe crystallization kinetics based on the mechanism of crystal nuclei formation, which applies to isothermal crystallization conditions; see Equation (5) above. However, the apparent Avrami index in the Jeziorny equation cannot explain the changes in crystal growth pattern and crystal dimension during non-isothermal crystallization. The Ozawa equation proposed by Ozawa is successful in describing the non-isothermal crystallization process of polymers with some simple conformations [49], but it cannot accurately describe other polymers and secondary crystallization in the crystallization process [50]. In recent years, Mo Zhishen combined the Avrami and Ozawa equations to propose a new model [51], namely the Mo Zhishen equation (referred to as the Mo equation), to more accurately describe the non-isothermal crystallization process of polymers (Equation (10)).

Combining the Avrami and Ozawa equations, based on a certain relative crystallinity ($X\left(t\right)$), Equation (10) is obtained.

(10) $$lgZ+nlgt=lgK\left(T\right)-mlg\phi$$

Let $F\left(T\right)={\left(\frac{K(T)}{Z}\right)}^{1/m},\alpha =\frac{n}{m}$, enter the above equation to obtain (11).

(11) $$lg\phi =lgF\left(T\right)-\alpha \cdot lgt$$

where t is time, min; n is the Avrami index, *m* is the Ozawa index, α is the ratio of the Avrami index to the Ozawa index, where m and n have the same physical meaning (as shown above); cooling rate is denoted by ϕ, °C/min; Z is the Avrami crystallization rate constant, min^−n^; $K\left(T\right)$ is a temperature-dependent cooling function, which is related to nucleation and crystal nuclear growth, ${\left(K/min\right)}^{m}$; and $F\left(T\right)$ is the cooling (heating) rate required to achieve a specific $X\left(t\right)$ in unit time [52], indicating the difficulty of crystallization of the material. For a certain relative crystallinity, the smaller $F\left(T\right)$ is, the faster the crystallization rate of the sample will be.

**Figure 10:**
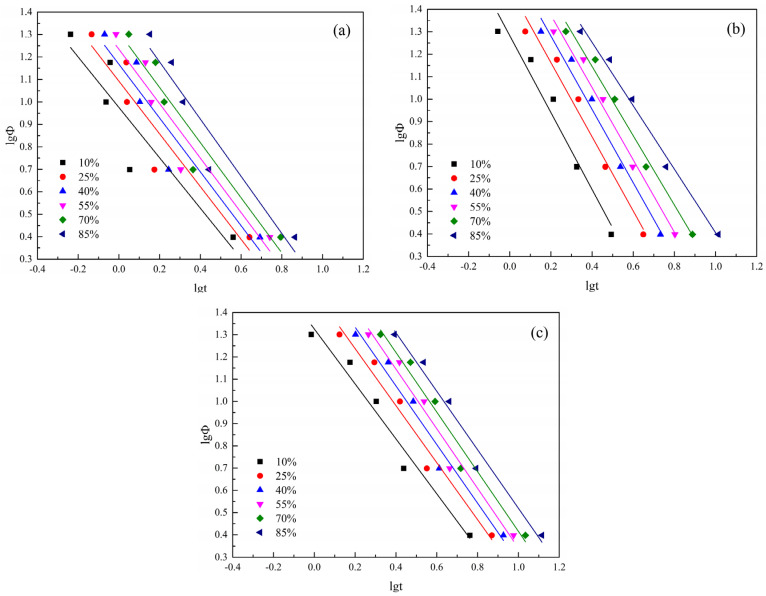
Relationship between lg*ϕ* and lg*t* at relative crystallinity of 10%, 25%, 40%, 55%, 70% and 85%. (**a**) S0; (**b**) S1.5; (**c**) S3.0.

According to Equation (11), the fitting curve of lgϕ vs. lgt was obtained at a certain $X\left(t\right)$. The slope of the curve is -α and the intercept is $lgF\left(T\right)$, see Figure 10, and the specific parameters of the curve are shown in Table 6.

α is the ratio of the Avrami and Ozawa indexes, n to m. It can be seen from Figure 10 combined with the data in Table 6 that under the same $X\left(t\right)$, R^2^ values of S1.5 and S3.0 are both greater than that of S0, which indicates that the data of S1.5 and S3.0 have a higher degree of agreement with Mo’s equation than that of S0. As can be seen from Table 6, *α* values of S0, S1.5, and S3.0 are all in the range of 1 to 2, which is consistent with the results in the literature [51].

**Figure 11:**
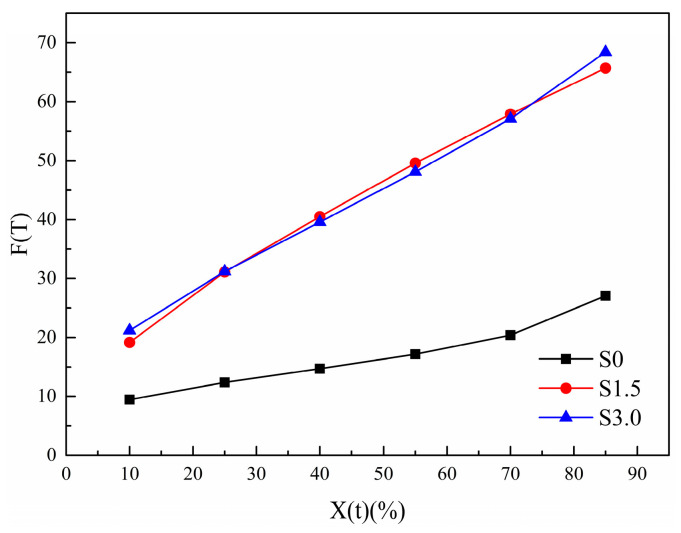
Change curve of Mo equation parameter *F*(*T*) of non-isothermal crystallization kinetics of PET and CDP with different SIPA contents.

The variation law of $F\left(T\right)$ with $X\left(t\right)$, in Table 6, is made into a curve, as shown in Figure 11. With the increase in $X\left(t\right)$, the $F\left(T\right)$ of all samples increases. This is because the crystal size increases with the increase in $X\left(t\right)$, resulting in an increased probability of extrusion and collision between spherical crystals. The required crystallization driving force increases and crystallization becomes difficult. As can be seen from Figure 11, under the same $X\left(t\right)$, $F\left(T\right)$ of S1.5 and S3.0 is larger than that of S0. This indicates that with the addition of SIPA, the non-isothermal crystallization of CDP molecular chains is slower than that of PET. This is because the meso-structure of SIPA in CDP and the sulfonate group lead to the destruction of the regularity of its molecular chain segments and an increase in random chain segments. At the same time, the ionic agglomeration effect formed by sulfonate ions obstructs CDP molecular chain migration, increasing the barrier to be overcome during CDP molecular chain crystallization, making crystallization more difficult, so $F\left(T\right)$ increases. However, the difference in $F\left(T\right)$ values between S1.5 and S3.0 is small, indicating that Mo’s equation makes it difficult to analyze the degree of crystallization difficulty of the two.

#### 3.2.8. Kissinger Method

The physical parameter of crystallization activation energy (△E) needs to be overcome during the crystallization migration of the polymer chain segment, to further investigate the non-isothermal crystallization behavior of cationic dyeable polyester with different SIPA contents. It was introduced to evaluate the crystallization capacity of the material. In this work, the Kissinger equation is used to obtain △E for data processing, as shown in Equation (12) [32].

(12) $$\frac{d\ln\left(\frac{\phi }{{T_{p}}^{2}}\right)}{d\left(\frac{1}{T_{p}}\right)}=-△E/R$$

where ϕ is the cooling rate at non-isothermal crystallization, °C/min; $T_{p}$ is the peak of the crystallization curve, that is, crystallization temperature, °C; △E is the crystallization activation energy, kJ/mol; and R is the gas constant with a value of 8.314 J/mol·K.

**Figure 12:**
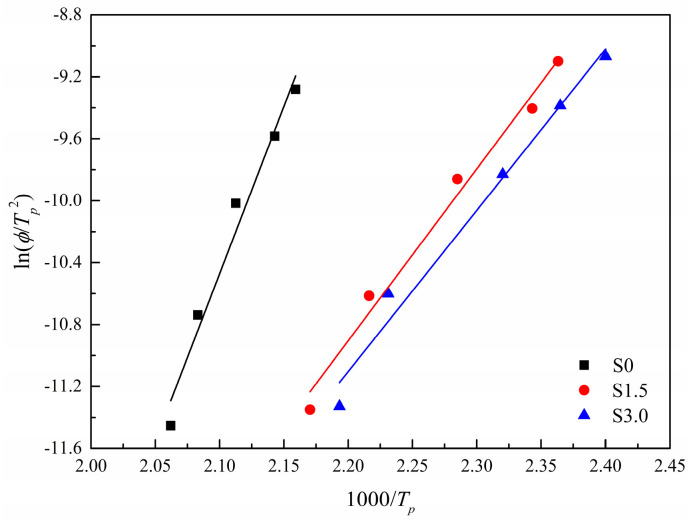
Kissinger plots of ln(*ϕ*/*T_p_*^2^) versus *Tp*^−1^.

The data were processed according to Equation (12) and the fitting curve of $ln(\frac{\phi }{{T_{p}}^{2}})$ and $\frac{1000}{T_{p}}$ can be obtained, as shown in Figure 12. The slope of the curve is −△E/(R×1000)), and its specific parameters are shown in Table 7.

It can be seen from Figure 12 and Table 7 that the absolute value of △E of S0, S1.5, and S3.0 gradually decreases. Since △E value also represents the energy released when polymer molecular chain segments are attached to the lattice during non-isothermal crystallization, the greater the absolute value of △E, the more molecular chain segments are discharged into the lattice and the easier crystallization is. Therefore, as the content of SIPA increases, crystallization becomes more and more difficult. This is because, on the one hand, the addition of SIPA destroys the regularity of CDP molecular chains and the sulfonate ion groups increase the steric hindrance between CDP molecular chains, and on the other hand, the sulfonate ion aggregates in CDP have a binding effect on CDP molecular chains. These two factors together lead to the increase in the barrier to overcome when CDP molecular chains are folded and orderly arranged. Therefore, the energy released by the crystallization of S1.5 and S3.0 is less, and the absolute value of △E of S1.5 and S3.0 is less than the absolute value of △E of S0. At the same time, when the content of SIPA increases, the barrier to be overcome in the non-isothermal crystallization of CDP molecular chains increases, and the difficulty of non-isothermal crystallization increases, so that the absolute value of △E of S3.0 is smaller than that of S1.5.

## 4. Conclusions

In this work, cationic dyeable polyester, CDP, was prepared by the SIPA one-step feeding method. The non-isothermal crystallization and kinetic behavior of PET and CDP with different SIPA contents have been investigated. A DCS test was performed at different cooling rates (2.5, 5, 10, 15, 20 °C/min), and the results show that the crystallization temperature of the sample gradually decreased with the increase in SIPA content. Compared with PET, the relaxation effect of the molecular chain has a more obvious effect on its crystallization with the increase in cooling rate, due to the ionic agglutination effect of sulfonate groups in CDP. The non-isothermal crystallization data of PET and CDP with different SIPA content were fitted by the Jeziorny equation, Ozawa equation, and Mo equation. The results show that with the increase in cooling rate, the driving force of crystallization increases, increasing the nucleation density and nucleation growth rate of PET and CDP. With the increase in SIPA content, the binding effect of sulfonate ion agglomeration on molecular chains in CDP is enhanced, and the nucleation density and crystal nuclear growth rate of CDP are decreased. The Ozawa equation is not suitable for the study of non-isothermal crystallization of CDP. These results are confirmed in the Kissinger equation. With the increase in SIPA content, the non-isothermal crystallization activation energy of the sample gradually decreases, making crystallization more difficult. The content of sulfonate has a significant influence on the non-isothermal crystallization behavior of CDP, which has a guiding effect on the development of the subsequent production process.

## Figures and Tables

**Table 1:** The intrinsic viscosity values of PET and CDP with different SIPA contents.

Sample	SIPA Content/mol%	[*η*]/dL·g^−1^
S0	0	0.681
S1.5	1.5	0.566
S3.0	3.0	0.494

**Table 2:** Parameters of PET and CDP crystallization at different cooling rates.

Sample	ϕ (°C/min)	$T_{b}$/°C	$T_{P}$/°C	$T_{e}$/°C	${△H}_{mc}$/(J/g)	ORC
S0	2.5	223.9	211.8	197.0	25.52	−0.802
	5	216.1	206.9	194.7	26.48	
	10	212.6	200.2	176.3	33.16	
	15	208.9	193.5	156.6	33.61	
	20	207.1	190.0	135.9	32.66	
S1.5	2.5	201.1	187.6	155.3	30.84	−0.465
	5	196.0	178.1	145.0	35.40	
	10	192.0	164.5	114.8	31.60	
	15	185.4	153.6	97.4	23.09	
	20	179.9	150.0	92.6	18.68	
S3.0	2.5	204.1	182.8	154.1	29.54	−0.446
	5	196.0	175.1	145.9	27.69	
	10	189.2	157.9	114.0	24.23	
	15	185.8	149.7	105.7	16.65	
	20	178.5	143.5	92.9	6.99	

**Table 3:** Melting temperature parameters of CDP and PET after non-isothermal crystallization.

Sample	ϕ (°C/min)	$T_{g}$/°C	$T_{m1}$/°C	$T_{m2}$/°C	$T_{cc}$/°C	${△H}_{cc}$/(J/g)	${△H}_{m}$/(J/g)
S0	2.5	79.5	245.0	/	/	/	39.60
	5	80.5	244.3	/	/	/	38.09
	10	79.9	242.7	249.8	/	/	37.46
	15	78.9	240.2	248.8	/	/	37.22
	20	81.6	239.1	249.8	/	/	36.80
S1.5	2.5	79.5	234.3	238.8	/	/	35.06
	5	79.7	/	240.4	/	/	37.01
	10	79.4	/	239.6	/	/	33.96
	15	78.4	/	239.3	157.4	2.68	38.80
	20	77.7	/	240.3	153.2	6.38	36.66
S3.0	2.5	81.5	231.6	237.5	/	/	33.68
	5	81.1	/	239.3	/	/	30.86
	10	79.7	/	238.8	/	/	35.92
	15	78.9	/	239.5	155.5	10.96	36.11
	20	78.5	/	239.7	157.1	18.65	35.58

**Table 4:** Non-isothermal crystallization kinetics by Jeziorny method.

Sample	*ϕ* (°C/min)	n_1_	$Z_{c1}$	R_1_^2^	n_2_	$Z_{c2}$	R_2_^2^
S0	2.5	3.450	0.131	0.966	2.882	0.206	0.965
	5	3.639	0.518	0.935	3.564	0.573	0.987
	10	2.400	0.871	0.931	2.929	0.822	0.951
	15	2.101	0.970	0.941	3.872	0.937	0.991
	20	1.703	0.995	0.954	3.043	0.976	0.976
S1.5	2.5	2.846	0.080	0.942	2.094	0.178	0.978
	5	2.784	0.409	0.932	2.520	0.464	0.988
	10	2.315	0.750	0.956	2.651	0.725	0.982
	15	2.328	0.878	0.984	2.666	0.841	0.984
	20	1.911	0.964	0.978	2.665	0.919	0.986
S3.0	2.5	3.523	0.032	0.945	2.636	0.076	0.970
	5	3.274	0.331	0.927	2.840	0.385	0.980
	10	2.961	0.676	0.973	2.541	0.694	0.963
	15	3.562	0.773	0.976	2.886	0.811	0.986
	20	2.525	0.918	0.936	2.563	0.908	0.989

**Table 5:** Non-isothermal crystallization kinetics by Ozawa method.

Sample	*T* (°C)	*m*	*lgk*(*T*)/(K/min)^m^	R^2^
S0	198	2.409	1.980	0.862
	200	2.741	2.020	0.851
	202	3.213	2.152	0.862
	204	3.825	2.341	0.876
	206	4.888	2.759	0.879
S1.5	164	1.902	1.499	0.919
	166	2.050	1.523	0.919
	168	2.218	1.557	0.919
	170	2.410	1.604	0.921
	172	2.640	1.667	0.922
	174	2.909	1.747	0.921
S3.0	158	1.854	1.573	0.954
	160	1.931	1.544	0.947
	162	2.035	1.538	0.943
	164	2.154	1.538	0.941
	166	2.283	1.541	0.940
	168	2.427	1.550	0.941

**Table 6:** Non-isothermal crystallization kinetics by the Mo method.

Sample	*X*(*t*) (%)	*α*	*F*(*T*)	R^2^
S0	10	1.122	9.45	0.805
	25	1.174	12.37	0.852
	40	1.198	14.71	0.872
	55	1.212	17.16	0.886
	70	1.227	20.40	0.895
	85	1.272	27.07	0.891
S1.5	10	1.712	19.17	0.950
	25	1.651	31.11	0.968
	40	1.633	40.50	0.975
	55	1.610	49.59	0.979
	70	1.542	57.89	0.983
	85	1.412	65.69	0.986
S3.0	10	1.235	21.18	0.960
	25	1.282	31.19	0.963
	40	1.319	39.57	0.964
	55	1.341	48.08	0.964
	70	1.341	57.09	0.965
	85	1.315	68.40	0.968

**Table 7:** The non-isothermal crystallization activation energy of PET and CDP with different SIPA contents by the Kissinger method.

Sample	ϕ/(°C·min)	$T_{p}$/°C	$△E/(\mathrm{kJ}\cdot {\mathrm{mol}}^{-1})$	$R^{2}$
S0	2.5	211.8	−179.7	0.967
	5	206.9		
	10	200.2		
	15	193.5		
	20	190.0		
S1.5	2.5	187.6	−92.3	0.983
	5	178.1		
	10	164.5		
	15	153.6		
	20	150.0		
S3.0	2.5	182.8	−86.5	0.977
	5	175.1		
	10	157.9		
	15	149.7		
	20	143.5		

## Data Availability

Data are contained within the article.
